# Freeze-Dried β-Glucan and Poly-γ-glutamic Acid: An Efficient Stabilizer to Strengthen Subgrades of Low Compressible Fine-Grained Soils with Varying Curing Periods

**DOI:** 10.3390/polym16111586

**Published:** 2024-06-03

**Authors:** Muralidaran Vishweshwaran, Evangelin Ramani Sujatha, Jair Arrieta Baldovino

**Affiliations:** 1 Centre for Advanced Research in Environment, School of Civil Engineering, SASTRA Deemed University, Thanjavur 613401, Tamil Nadu, India; vishweshwaran36@gmail.com; 2 Applied Geotechnical Research Group, Department of Civil Engineering, Universidad de Cartagena, Cartagena de Indias 130015, Colombia

**Keywords:** freeze-drying, β-glucan, poly-γ-glutamic acid, biopolymer, geotechnical, CBR

## Abstract

The freeze-drying of biopolymers presents a fresh option with greater potential for application in soil subgrade stabilization. A freeze-dried combination of β-glucan (BG) and γ-poly-glutamic acid (GPA) biopolymers was used to treat low compressible clay (CL) and low compressible silt (ML) soils in dosages of 0.5%, 1%, 1.5%, and 2%. The California bearing ratio (CBR) test for the treated specimens was performed under three curing conditions: (i) thermal curing at 60 °C, (ii) air-curing for seven days followed by submergence for 4 days, and (iii) no curing, i.e., tested immediately after mixing. To investigate the influence of shear strength on the freeze-dried biopolymer-stabilized soil specimens and their variations with aging, unconfined compressive strength (UCS) tests were conducted after thermal curing at 60 °C for 3 days, 7 days, and 7 days of thermal curing followed by 21 days of air curing. The maximum CBR of 125.3% was observed for thermally cured CL and a minimum CBR of 6.1% was observed under soaked curing conditions for ML soils. Scanning electron microscopy (SEM), infrared spectroscopy, average particle size, permeability, and adsorption tests revealed the pore filling, biopolymer adsorption and coating on the soil surface, and agglomeration of the soil along with the presence of hydrogen bonds, covalent amide bonds, and Van der Waals forces that contributed to the stiffening of the stabilized soil. Using three-dimensional (3D) finite element analysis (FEA) and layered elastic analysis (LEA), a mechanistic–empirical pavement design was carried out for the stabilized soil and a design thickness catalog was prepared for the maximum CBR. The cost reductions for a 1 km section of the pavement were expected to be 12.5%.

## 1. Introduction

Highway infrastructure is an essential component in the growth and development of a nation since it facilitates connectivity and accessibility by providing ease of access. One of the most important elements that contribute to the deformation of pavements is the failure of the supporting subgrade. It is impossible for large deposits of clays that have weak geotechnical properties to withstand traffic loads because the subgrade strength is not sufficient [1]. Weak, fine-grained soil presents an engineering barrier that must be overcome to construct pavements that are both effective and long-lasting. Some of the issues that arise when facing problematic soils for use as a subgrade include the rising costs of construction materials, the pollution that is caused as a result of using these materials, and the lack of availability of neighboring soils that exhibit appropriate engineering features. The installation of a costly deep foundation or the restoration of soil are not the only options for soils that have inherently inadequate engineering qualities. It is vital to select an additive that is environmentally friendly, economical, and non-toxic and that can improve the engineering features of the soil through its application [2]. Sustainable soil stabilization contributes to overall environmental stability by reducing runoff, improving water quality, mitigating climate change impacts, and supporting sustainable land use practices. Geotechnical engineering projects often involve the extraction and use of natural resources such as soil, rock, and water. Sustainable practices aim to minimize resource consumption, optimize material usage, and promote recycling and reuse wherever possible, which reduces the depletion of finite resources and minimizes the environmental footprint of construction activities [3]. An environmentally responsible alternative for enhancing the ground is to use biological approaches.

Biopolymers are naturally occurring monomeric units that repeat and can be found in plants, animals, and microorganisms. Because of the molecular structure of these substances, they can form gels and networks that are interconnected [4]. They have a high molecular weight and are capable of producing hydrogels according to their properties. Microorganisms, plants, and animals are some of the common sources from which the diverse polysaccharide and protein biopolymers originate. Biopolymers, with their numerous functional groups, are well-suited to a wide range of industrial and medical applications [5]. The addition of lignin to CL soil resulted in a reduction in the effective angle of shearing resistance and an enhancement of effective cohesion by 22.1% and 120.8% [6]. Lemboye and Almajed [7] improved the UCS of pectin-stabilized sandy soil by 137-fold and attributed the strength gain to the loss in the moisture of the stabilized sand. Hamza et al. [8] evaluated the long-term strengthening effect of a problematic swelling clay on treatment with guar gum biopolymer and noted a linear increase in strength with the curing period, with the UCS attaining 654 kN/m^2^ after 365 days. Babatunde and Byun [9] treated sandy soil with zein biopolymer and enhanced its stiffness by 2050%. Martin et al. [10] recommended the usage of enzyme-induced carbonate precipitation (EICP) for bio-cementitious soil columns for field applications due to its fulfillment of the targeted strength of 0.5 MN/m^2^. Liu et al. [11] observed a 98.8% reduction in the wind erosion resistance and an 800% increase in the cone penetration resistance of sand subjected to the microbial-induced carbonate precipitation (MICP) technique. Reddy et al. [12] noted increases in the liquid and plastic limits of a bio-char-stabilized soil of 25.8% and 42.5%, respectively, while the coefficient of permeability was reduced by 4086%.

By cross-linking two different biopolymers, it is possible to create a stable polymer structure in three dimensions, which improves the mechanical properties of the composite biopolymer [13]. Recent studies from the last decade have revealed that combining a biopolymer with another biopolymer, natural fibers, MICP, or another additive results in improvements in the mechanical behavior of soils. Meng et al. [14] highlighted improved hydrogen bonding and an enhanced soil–water retention ratio by the synergistic effects of lignin and polysaccharides. Khatami and Kelly [15] highlighted a synergistic influence on the reduction in grout bleeding by 50% and 98%, respectively, upon combining xanthan gum with guar gum and cellulose. Ren et al. [16] emphasized an improvement in the soaked UCS of combined xanthan gum–casein biopolymers of 612% after immersing the soil specimen in water for 24 h. Ni et al. [17] combined carrageenan and casein biopolymers in the stabilization of a low compressible clay soil, resulting in improvements in the coefficient of consolidation and UCS of 60% and 155%, respectively. Sorze et al. [18] emphasized the synergistic capabilities and hydrogen bonding of cellulose fibers in forming an interconnected network with xanthan gum biopolymer for soil stabilization. Ramachandran et al. [19] noted the synergistic influence of xanthan gum and MICP, achieving the targeted superior strength of low compressible soil in addition to a reduction in the release of ammonia compared to the standalone MICP process. Ma et al. [20] and Chen et al. [21] noted the synergistic interaction of xanthan gum with natural fibers in reducing the brittleness and increasing the tensile strength of soil specimens. Feng et al. combined xanthan gum with jute fiber and enhanced the maximum split tensile strength and UCS by 3300% and 3440%, respectively, for residual moisture contents of 100% and 0%, respectively [22].

The biopolymers utilized in this study included BG and poly-γ-glutamic acid (GPA). BG is classified as a polysaccharide, while GPA is categorized as an amino acid biopolymer. D-glucopyranosyl residues are the building blocks of BG, which are linked together by glycosidic linkages [23]. GPA, being amphiphilic, can interact with both hydrophobic and hydrophilic substances, allowing for versatile applications, and was synthesized by microbial fermentation [24]. Moradi et al. [25] improved the CBR of problematic clay soil from 46% to 101% upon stabilization with BG. Soldo et al. [26] recorded that the 31% tensile strength improvement in BG-treated silty sand was the highest when compared to xanthan gum, guar gum, chitosan, and alginate biopolymers. Yao et al. [27] demonstrated the capability of γ-polyglutamate in improving the MICP, which resulted in a 260% increase in the UCS of coarse soil. In a study conducted by Khachatoorian et al. [28], the coefficient of permeability of sand was lowered by a factor of one million after 11 days of curing. The above literature review reveals the efficacy of BG and GPA in ground improvement. However, very few studies have been conducted on the capability of GPA and BG in subgrade strengthening, and the freeze-drying process of these two biopolymers in CL and ML soil stabilization has not been researched before, to the best of the author’s knowledge. Hence, in this study, the effect of freeze-dried BG and GPA on select geotechnical properties like the liquid and plastic limits, compaction behavior, unconfined compressive strength, and subgrade strength of two low compressible soils, CL and ML, was investigated.

## 2. Materials and Methods

### 2.1. Materials

The two fine-grained soils were collected from Srirangam, Trichy district, at the locations 10°52′34.6″ N 78°38′30.8″ E and 10°52′32.7″ N 78°38′20.5″ E, respectively. The two in situ soils were extracted at a depth of two meters to avoid loose unconsolidated deposits on the surface and vegetative debris. The organic content of the soils was less than 3%. Geotechnical tests were conducted for the oven-dried soils, and the soils fell into the CL and ML categories, according to the unified soil classification system (USCS). The UCS of the two soils shown in Table 1 indicates that they possessed stiff and very stiff consistencies.

**Figure 1:**
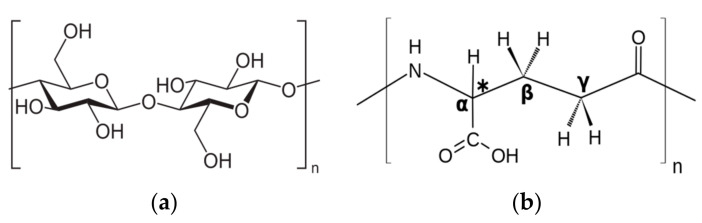
Chemical composition: (**a**) BG; (**b**) GPA.

BG biopolymer was purchased from Meteoric Life Sciences, Ahmedabad, GJ, India, and GPA was purchased from Zytex Biotech Private Limited, Mumbai, MH, India. They are both natural polymers that possess distinct structures, functional groups, production processes, and applications in diverse industries. BG is predominantly derived from natural sources such as fungus, oats, barley, and seaweed. It can also be produced through enzymatic synthesis [29]. The primary functional groups present in GPA are carboxyl (-COOH) and amide (-CONH) groups, which influence its water solubility and biocompatibility [30,31]. Figure 1 depicts the chemical composition of BG as well as GPA.

### 2.2. Methods

**Figure 2:**
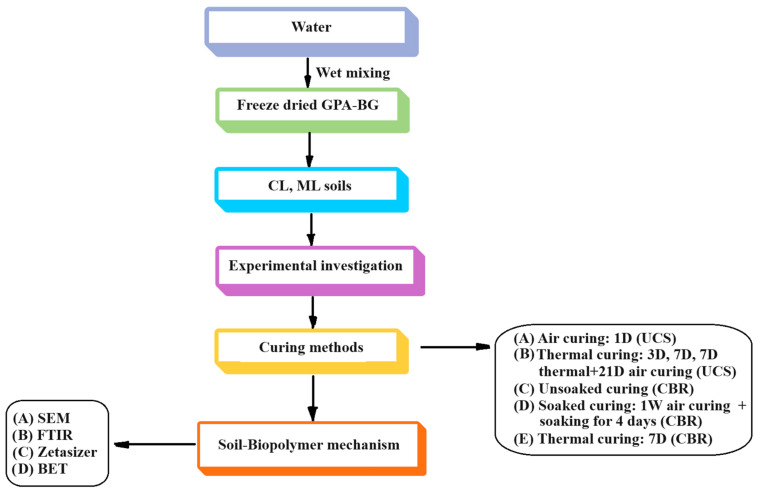
Methodology.

Figure 2 shows the methodology used.

Both the biopolymers were thoroughly mixed and then subjected to freeze-drying at a temperature of −1.5 °C for 72 h [32,33]. It was observed by Rezaeimalek [34] that wet mixing facilitated the coating of biopolymers onto the surface of soil, and its effectiveness on the strength performance was discovered to be superior to that of dry mixing. Therefore, the biopolymer solution was generated by adding the necessary quantity of water to the powdered form, and then the solution was kept sealed with wraps for a period of two hours. After that, the biopolymer solution was combined with the soil by first combining the soil with 1% water, and then, immediately after that, the biopolymer solution was mixed with the soil to complete the process. In order to prevent the soil mixture from losing its moisture, it was sealed for a period of two hours. After the soil had reached an equilibrium moisture content, it was molded in order to conduct an evaluation of its geotechnical qualities. Following a sample preparation period of two hours, the UCS of both the untreated and treated soil was calculated immediately after taking the samples. In order to estimate the UCS, thermal curing at a temperature of 60 °C was also utilized for three days, seven days, and seven days of thermal curing, in addition to air curing for twenty-one days.

The CBR specimens were air-dried over 7 days and then immersed in water for 4 days. The test was also conducted under unsoaked conditions instantaneously after sample preparation and thermal curing at 60 °C for a 7 d period. TESCAN VEGA3 SEM was utilized to observe the soil aggregate structure of the control and freeze-dried GPA-BG-stabilized soils. A Malvern zetasizer particle size analyzer was used to determine the average particle diameter of the soils and a Brunauer–Emmett–Teller (N_2_-BET) analyzer gas adsorption test was used for nitrogen adsorption analysis. The experiments presented were carried out conforming to the prescribed methods to estimate the select index and engineering properties of the control and GPA-BG-stabilized soils. With reference to IS: 2720 Part 5 (1985) [35] from the Bureau of Indian Standards (BIS), tests to determine the Atterberg’s limit were conducted. The light compaction test that conforms to IS: 2720 Part 7 (1980) [36] was performed during this study. The UCS test was conducted following guidelines presented in IS: 2720 Part 10 (1991) [37]. The CBR test according to IS: 2720 Part 16 (1987) [38] was conducted to determine if the modified soils would be feasible as a subgrade layer. A permeability test was performed with a falling head in accordance with the requirements of IS 2720 Part 17 (1986) [39]. FTIR analysis was performed with a Bruker Alpha instrument and the number of scans in the test was 24 at a 4 cm^−1^ resolution. The ASAP2020, Micromeritics, Norcross, GA, USA instrument was used for the determination of specific surface area (SSA). The SSA analysis for bath temperature and equilibration interval were −195 °C and 5 s, respectively. Through spectroscopic and microscopic studies, the bonds related to the interaction between the biopolymers could be identified as the key factor behind the changes in the overall properties. Loads on the pavement and material properties of the flexible pavement modeling were adopted, conforming to Indian Roads Congress (IRC) [40].

Stretching and bending were the commonly observed modes of IR interactions in the FTIR experiment. The number of scans amounted to 23. With reference to IS: 2720 Part 5 (1985) from the Bureau of Indian Standards (BIS), the tests to determine the Atterberg’s limit were conducted thrice and the average of the three was adopted. The light compaction test that conformed to IS: 2720 Part 7 (1980) was performed thrice and the average of the three was adopted. The UCS test was conducted following guidelines presented in IS: 2720 Part 10 (1991) and the average of the three trials was adopted. The CBR test according to IS: 2720 Part 16 (1987) was conducted to determine if the modified soils would be feasible as a subgrade layer and the average of the three trials was adopted. A permeability test was performed thrice with a falling head in accordance with the requirements of IS 2720 Part 17 (1986). FTIR was performed twice, whereas SEM, BET, and zetasizer tests were performed once.

## 3. Results

### 3.1. Atterberg’s Limits

**Figure 3:**
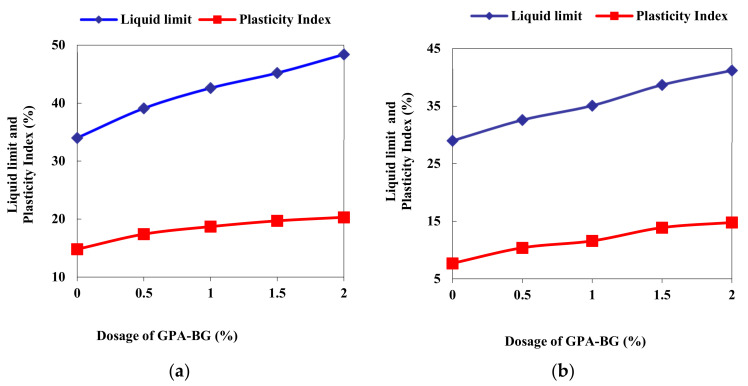
Liquid limit and plasticity index: (**a**) GPA-BG-stabilized CL; (**b**) GPA-BG-stabilized ML.

The freeze-dried GPA-BG biopolymer caused the liquid limit and plasticity index to increase as their dosage increased. Maximum liquid limits of 48.4% and 41.2% and maximum plasticity indices of 20.3% and 14.8% were observed for the 2% dosage of GPA-BG in CL and ML soils. The hydrophilic nature of the biopolymer led to increased water absorption with higher dosages, resulting in a higher plasticity index. Although BG and GPA solutions did not form instantaneous thick gels, the treated soils showed a viscous, firm texture when mixed during the liquid and plastic limit determination processes. The particular surface of the soil and the double layer of the soil both had an effect on the liquid limit of the soil itself [41]. The adsorption of the biopolymer by the soil occurred as a result of hydrogen bonding, which gave rise to variations in the Atterberg’s limits shown in Figure 3.

### 3.2. Compaction Characteristics

**Figure 4:**
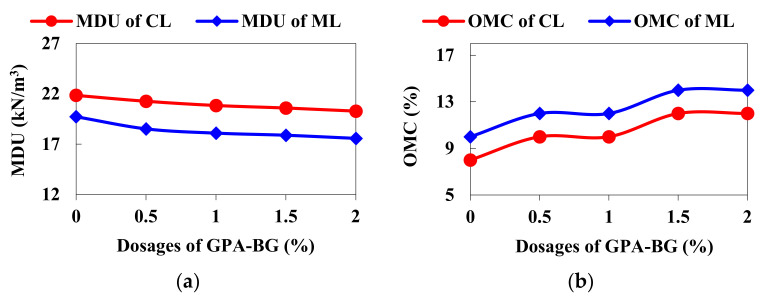
Standard proctor test curves: (**a**) MDU of GPA-BG-stabilized soils; (**b**) OMC of GPA-BG-stabilized soils.

The MDU of the GPA-BG-stabilized CL and ML reduced to 20.28 kN/m^3^ and 17.58 kN/m^3^, respectively, for the 2% dosage. The OMC for the same increased to a maximum of 12% and 14% for the maximum dosage, as shown in Figure 4. The excess water addition directly affected the MDU of the stabilized soils at all dosages. The freeze-dried GPA-BG mixture possessed lower specific gravity compared to the CL and ML soils, which affected the MDU of both soils. The GPA-BG additive consumed additional water due to the water-imbibing property of BG and the filling of pores. This had an impact on the clay’s double layer, resulting in the treated clays needing more water. Although the biopolymer solution initially bonded with the soil, it eventually caused the soil particles to move further apart as the water content increased, affecting workability.

### 3.3. UCS

**Figure 5:**
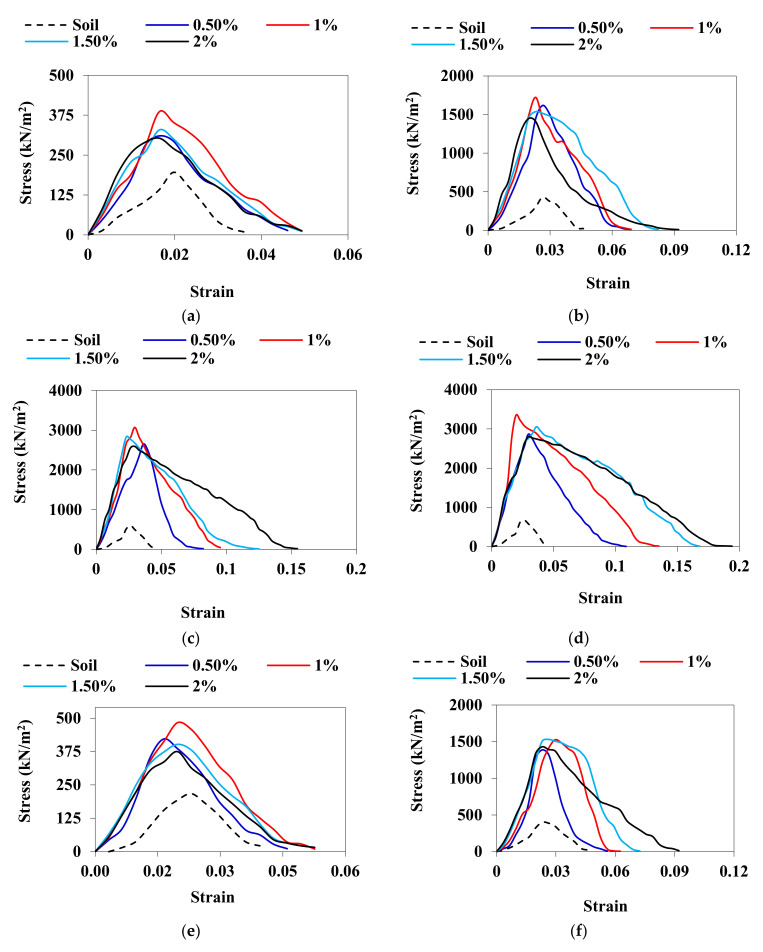

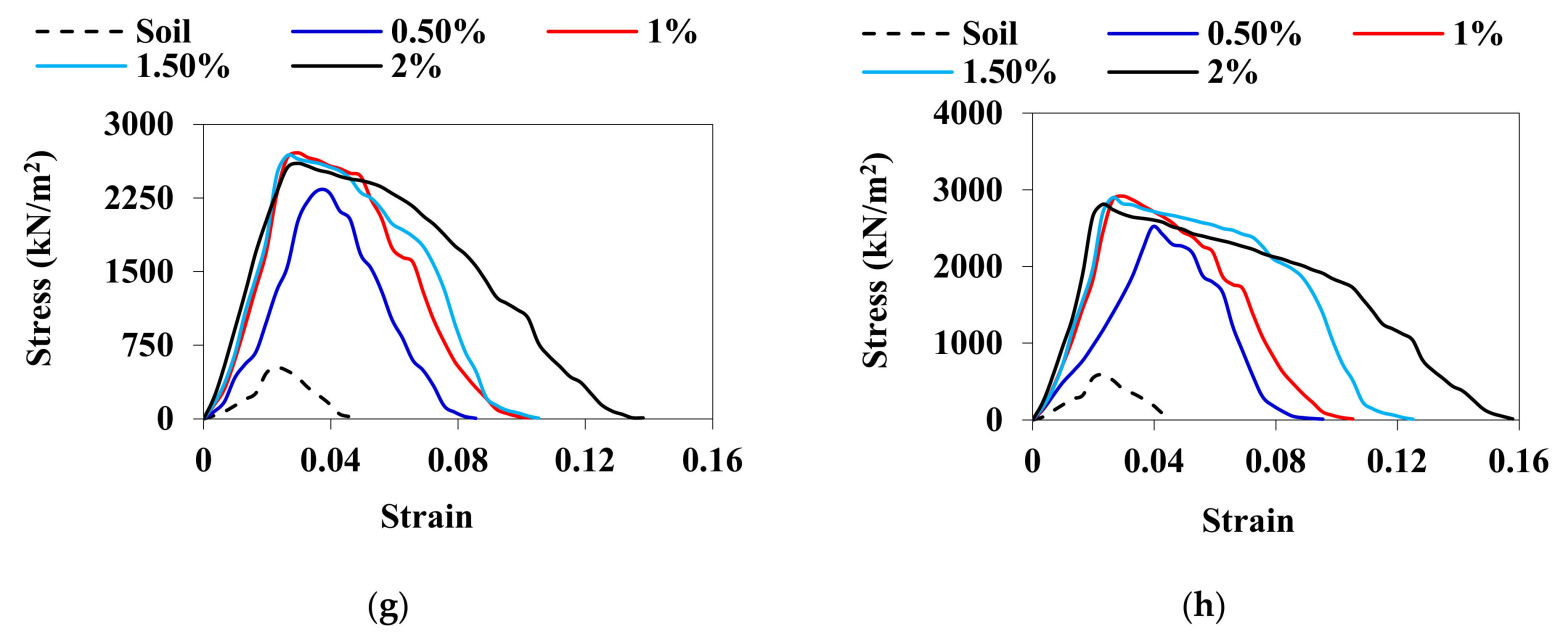
UCS test curves: (**a**) GPA-BG–CL: 2 h; (**b**) GPA-BG–CL: 3 days; (**c**) GPA-BG–CL: 7 days; (**d**) GPA-BG–CL: 28 days; (**e**) GPA-BG–ML: 2 h; (**f**) GPA-BG–ML: 3 days; (**g**) GPA-BG–ML: 7 days; (**h**) GPA-BG–ML: 28 days.

**Figure 6:**
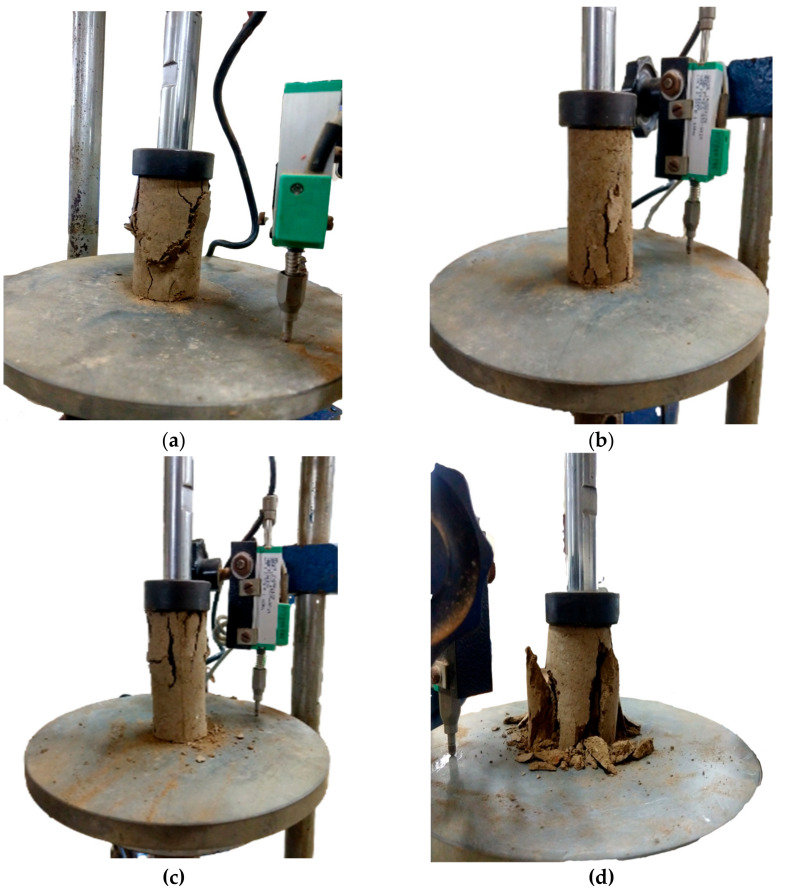
UCS failure specimens: (**a**) GPA-BG–CL: 2 h; (**b**) GPA-BG–CL: 28 days; (**c**) GPA-BG–ML: 2 h; (**d**) GPA-BG–ML: 28 days.

After 2 h of sample preparation, the UCS of GPA-BG-treated ML and CL soils was measured as 483 kN/m^2^ and 386 kN/m^2^, respectively. The untreated CL and ML soils that were not treated thermally showed a UCS of 316 kN/m^2^ and 268 kN/m^2^, respectively. The highest UCS was achieved through thermal curing of the two soils. The CL soil showed the highest UCS after 28 days of curing, reaching 3349 kN/m^2^ with a 1% addition of GPA-BG biopolymer. GPA-BG-stabilized CL showed a noticeable 911% maximum rise in UCS during the initial 7 days of the cured specimens, in contrast to the rate of strength gain from 7 days to 28 days. The stress–strain behavior of untreated and treated soils and the failure mode are shown in Figure 5 and Figure 6, respectively.

Figure 5 reveals the brittle failure of both the CL and ML soils stabilized by GPA-BG after 28 days of curing, while shear failure was observed after 2 h of sample preparation. The biopolymer solution was transformed into filamentous strands as a result of the loss of water that occurred during the drying process. After being tested for two hours, the UCS of the specimens displayed no dramatic peaks at failure and did not reveal any signs of brittleness. During thermal treatment, the decrease in unconfined compressive strength (UCS) was less pronounced for soils with larger dosages of biopolymers compared to soils that were not thermally treated. Chang et al. [42] showed that submerging a thermo-gelation biopolymer-treated soil after air drying improved its strength when compared to untreated soil, even after being submerged. Unprocessed soils do not experience significant deformation even after undergoing heat treatment. A non-linear increase in the stiffness of the GPA-BG-stabilized soils was observed in the air-cured specimens that were examined after two hours.

### 3.4. CBR

**Figure 7:**
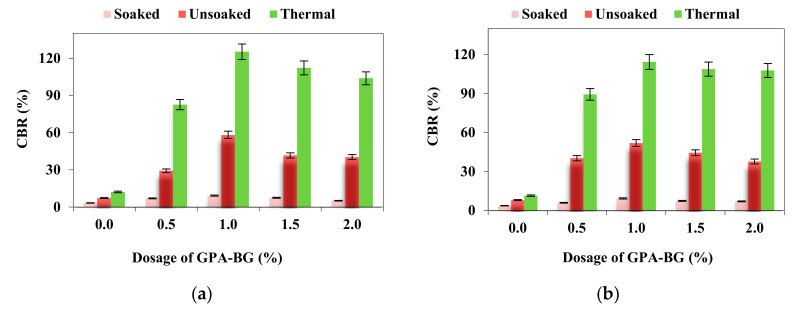
CBR under varying curing types: (**a**) GPA-BG-stabilized CL; (**b**) GPA-BG-stabilized ML.

The maximum CBR for the soaked, unsoaked, and thermally cured 1% GPA-BG-stabilized ML specimens was found to be 9.38%, 52.05%, and 114.41%, respectively. The flexible pavement design was based on the worst-case situation, which was the soaked state, as defined by IRC [40]. Figure 7 reveals that both control soils were not suitable for the subgrade preparation of flexible pavement.

The method of curing influenced the CBR of the soil for both unstabilized and GPA-BG-stabilized soils. Allowing the specimens to air-cure for a week or two before putting them through testing enabled the treated soil to offer enhanced penetration resistance. Both air-curing and thermal curing at 60 °C result in a more compact and rigid clay–polymer matrix. A significant 84% and 92% decrease in CBR was seen for specimens that were submerged compared to those that were unsoaked and thermally cured, respectively. The duration of the curing process improved the tangent modulus of the GPA-BG-stabilized CL and ML soils. A cost reduction of 12.5% was noted for the GPA-BG-stabilized CL soil under a 20 million standard axle (MSA) traffic intensity over a 1 km stretch of flexible pavement. This reduction in cost was due to the reduction in thickness of the top bituminous surfacing and the granular layer from 220 mm to 190 mm and from 245 mm to 220 mm, respectively. Khaleghi and Masood [43] and Gopal and Chamberlin [44] reported 284% and 250% increases in the soaked CBR of sand and clay soils by the adoption of zein and xanthan gum biopolymers, respectively. A design thickness catalog was prepared for the best-performing CBR based on the recommendations of IRC [40] and is presented in Table 2. This design of the flexible pavement was carried out by 3D FEA and LEA using EverStressFE 1.0 and KenLayer 2.0 software.

### 3.5. Fourier-Transform Infrared Spectroscopy (FTIR)

**Figure 8:**
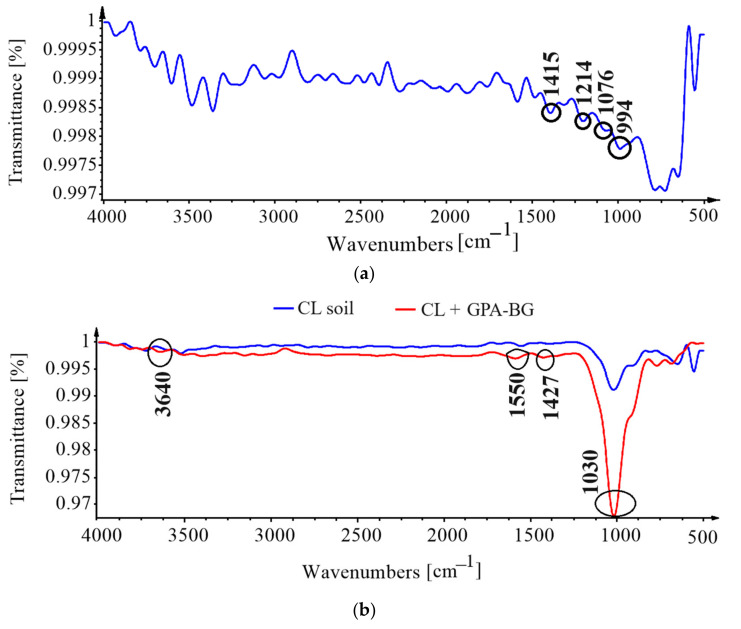

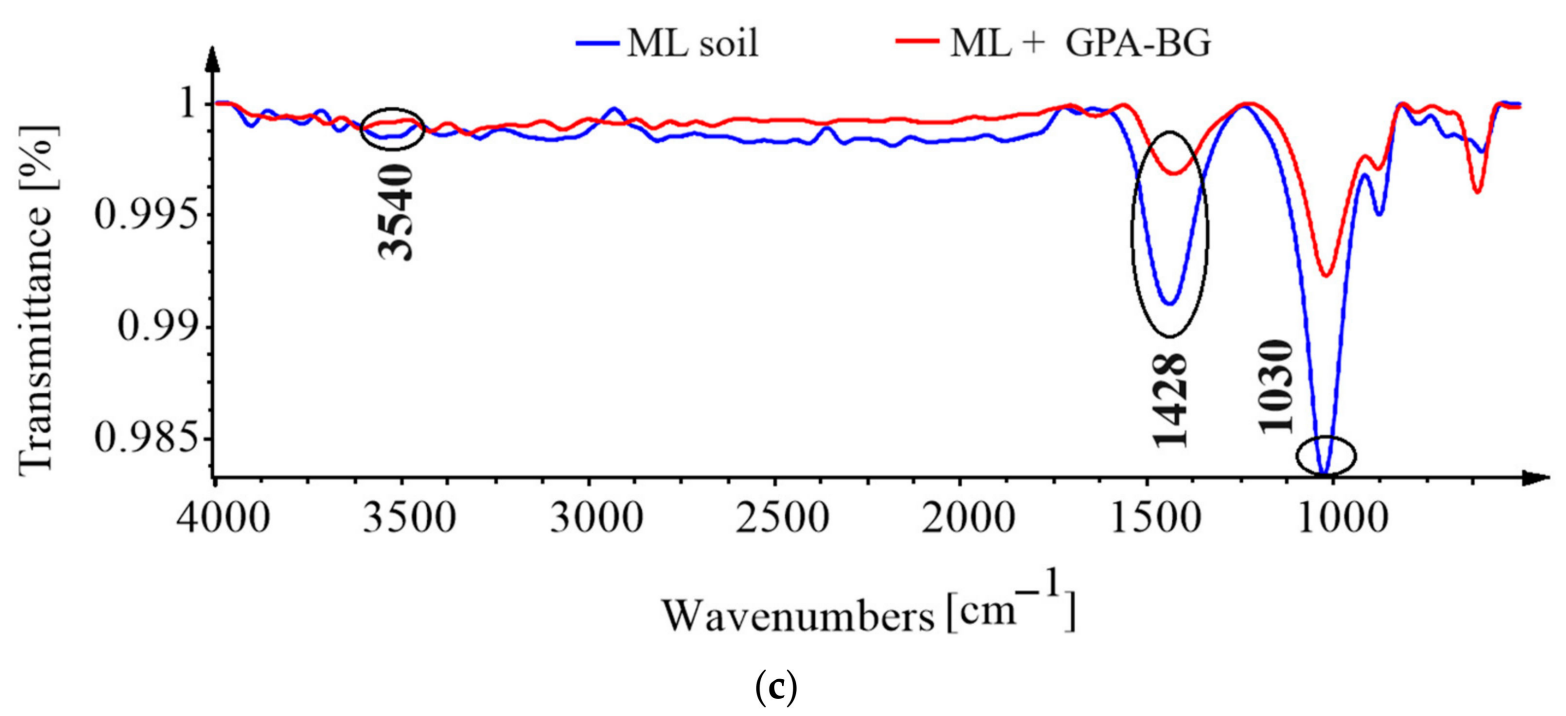
FTIR spectra: (**a**) freeze-dried GPA-PG; (**b**) GPA-BG-stabilized CL; (**c**) GPA-BG-stabilized ML.

The characteristic bands of the spectra that represent the wavenumbers that are displayed in Figure 8 are demonstrated by the corresponding wavenumbers that resulted from the molecular vibrations that occurred as a result of the interaction between GPA-BG and ML and CL soils.

The FTIR spectra of freeze-dried GPA-BG biopolymer revealed the presence of BG by the peaks corresponding to the wavenumbers 994 cm^−1^ and 1076 cm^−1^. The peaks of GPA were at 1214 cm^−1^ and 1415 cm^−1^ [45,46]. As indicated in Figure 8, there existed a sharp increase in intensity at 1030 cm^−1^ for both CL and ML. The characteristic vibration modes were the stretching of Si-O and the stretching of Si-O-Si for the functional groups of clay minerals and silica, respectively [47]. In addition, the increase in intensity could facilitate an enhanced interaction of the CL and ML soils with the GPA-PG, leading to adsorption, bonding, and the formation of hydrated stiffened complexes by the fulfillment of voids. The stretching of Si-OH and OH was observed for both the soils’ wavenumbers at 3540 and 3640, respectively, emphasizing the hydrogen bonding of the soil–biopolymer treatment [47]. The wavenumber 1428 cm^−1^ with increasing intensity indicated the COO^-^ stretching of carboxylic acid as well as CH_2_ scissoring. The bending vibration of NH and stretching vibration of CN were also observed for the amide bond with the ML soil [48].

### 3.6. SEM Analysis

**Figure 9:**
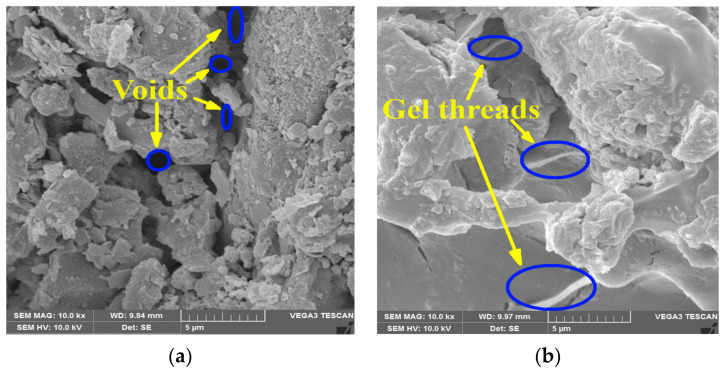
SEM results after 28 days of failed UCS specimen: (**a**) CL soil; (**b**) GPA-BG-stabilized CL.

According to the illustration in Figure 9a, the pore gaps in the soils could have been the result of inadequate hydration and the loose packing of individual grains. Figure 9b demonstrates the structural alteration that occurred as a result of treatment with biopolymers.

Through the process of drying the soils and increasing the number of curing days, the soil matrix was strengthened as a result of the stiffening of the fiber connections. The GPA-BG biopolymer underwent solidification, causing the clay particles to cluster together and enhancing the chemical bonds formed through the interaction between the clay and the polymer [49]. The gel threads composed of GPA-BG enhanced the rigidity of the soil matrix, thus enhancing its strength. Furthermore, the GPA biopolymer not only filled empty spaces but also adhered to the surface of clay soils through molecular affinity [50]. This phenomenon has been observed and documented in studies. BG with high tensile strength has the ability to prevent the formation of cracks [26]. BG formed hydrogen bonds with the soil particles and formed a coating on the surface of the stabilized soils [51]. The presence of amide bonds caused the soil–GPA-BG complex to clump together, as evidenced by several threads interweaving throughout the soil structure [27]. The soil-biopolymer connections remained intact after 7 days of air-drying and 21 days of air-drying.

### 3.7. Zetasizer and BET Analyses

**Figure 10:**
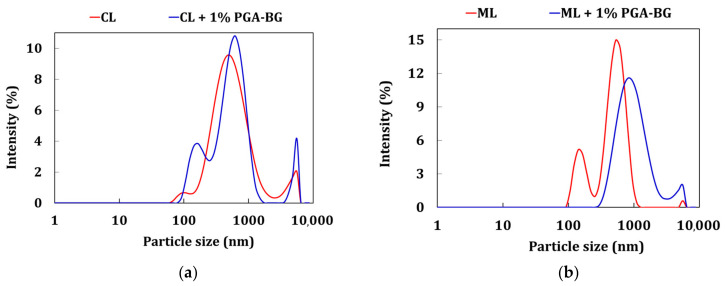
Zetasizer particle size: (**a**) GPA-BG-stabilized CL; (**b**) GPA-BG-stabilized ML.

The CL and ML soils had particle sizes of 473.7 nm and 610 nm, respectively. The GPA-BG-treated ML showed a 38% increase in average particle size compared to the control soil. After 28 days of air-curing, both the CL and ML soils underwent agglomeration and flocculated upon the addition of GPA-BG. GPA-BG-treated soils adsorbed onto the clay surface, and clay particles tended to cluster at important zones within the clay–biopolymer complex, which led to an expansion in particle size. The BET findings demonstrated that the addition of GPA-BG to the fine soil resulted in a 42% decrease in the SSA of the CL soil, as similarly observed by Latifi et al. [49]. In accordance with the findings of the SEM and FTIR investigations, which demonstrated that the biopolymer had good adhesion to the clay minerals, this finding is consistent. The results of the average particle size of the soils are shown in Figure 10.

## 4. Discussion

BG, renowned for its ability to form films and exhibit viscoelastic qualities, functions as a strengthening agent when paired with GPA [52]. BG is highly crystalline and its micro-crystallinity zones can also serve as a physical network, connecting points by hydrogen bonding [33]. The biopolymer network has the ability to occupy empty areas within a soil matrix, thus improving the cohesion and stability of the soils. Electrostatic attraction and surface adhesion in silty soils lead to the aggregation and stability of particles, thus inhibiting the dispersion of silty soil under soaked conditions [53]. Freeze-drying induces the formation of a porous framework in the GPA-BG blend, thus augmenting the surface area, promoting soil grains to occupy nearer positions, and enhancing the soil stiffness [54]. The freeze-dried GPA-BG promoted physical adsorption and hydrogen bonding with silt particles due to its larger surface area and enhanced reactivity. The GPA-BG biopolymer could adhere to the clay soil by means of adsorption and surface contact processes. The freeze-drying technique enhanced the formation of intermolecular entanglement and crosslinking, resulting in a robust and linked structure with the stabilized clay [33,55]. Hydrogen bonds were established between the hydroxyl groups of the biopolymers and the surfaces of clay minerals, thus enhancing adhesion. The hydrophilic characteristic of the GPA-BG in clay soils promoted bonding with clay particles, thus increasing cohesion [56]. Ionic interactions were formed between the cations in the clay soil and the carboxyl groups with a negative charge in the GPA-BG biopolymer. Clay particles had a tendency to flocculate at important points in the clay–biopolymer complexes, which led to an increase in particle diameter. According to Taniguchi et al. [57], bundled clay clusters are not caused by the viscosity of the bipolymer but rather by the absorption of water.

The inclusion of nitrogen in the GPA-BG biopolymer enhanced the long-term stability of the soil matrix biochemically [58]. Compared to the submerged situation, the frictional resistance was greater under dry conditions, which indicated that the barrier to penetration was higher under dried conditions [50]. It was determined that the microstructural changes that occurred over the curing days resulted in an increase in the soil’s resistance to penetration. The agglomeration of the GPA-BG biopolymer occurred as a result of its ability to move and interact with other molecules in the biopolymer structure, resulting in the formation of stable regions [49]. When the dosage of BG was too high, it caused the clay particles to be pushed, resulting in a decrease in electrostatic bonding. As the percentage of biopolymers in the GPA-BG rose, the liquid and plastic limits also increased due to the hydrophilic attribute of biopolymers for gel formation. Although the GPA-BG solution bonded with the soil, it eventually caused the soil particles to separate as the water content increased [59]. The hydrophilic biopolymer had the ability to soak up water from the empty spaces in the soil, which had an impact on the clay’s double layer, resulting in the treated clays needing more water. The additional OMC coupled with the reduced specific gravity of the GPA and BG biopolymers resulted in the inverse relationship between the OMC and MDU [60]. The linear drop in the coefficient of permeability with the increase in GPA-BG dosages from 7.31 × 10^−5^ cm/s to 4.77 × 10^−6^ cm/s highlighted its sealing characteristics owing to the repletion of the gaps in the soil. The bonding processes in the GPA-BG-stabilized soils consisted of a combination of physical interactions, such as hydrogen bonding and electrostatic interactions, as well as chemical bindings, such as covalent bonds. Consistent increases in FTIR transmittance at various peaks in addition to critical shifts in the spectra indicated the efficacy of the GPA-BG additive in inducing modifications to the structure of the soil.

Table 3 below shows a cost comparison of unstabilized and GPA-BG-stabilized pavement. A design thickness catalog was used for the GPA-BG-stabilized pavement and empirical design thickness was adopted for the control ML soil. The cost comparison was made for a traffic intensity of 20 million standard axles (MSA) over a length of 1 km and width of 3.75 mm. The cost of the GPA and BG biopolymers is INR 250/kg.

## 5. Conclusions

Based on the results and discussion, it is observed that the thermally treated GPA-BG-stabilized CL and ML soils recorded a maximum UCS of 3349 kN/m^2^ compared to air-cured soils. The contrast in CBR values between soaked curing with air- and thermal curing reveals that the biopolymer is highly suitable for arid, low-rainfall regions or pavements requiring temporary support. Despite air-curing for 7 days, the submergence of the soil sample reduced the CBR by 92%. Freeze-drying the GPA-BG strengthened the CL and ML soils by adhesion, amide bonds, and hydrogen bonds. The filling of pores and thermosetting contributed to increasing the strength of the stabilized soils. Curing time promoted flocculation, stiffening, and greater strength gain in addition to the filling of voids. A cost reduction of 12.56% is achievable for a 1 km stretch of the GPA-BG-stabilized ML soil flexible pavement due to the reduced thicknesses of the bituminous and granular layers.

The usage of natural soil for infrastructure development is vital for sustainability in construction and the upkeep of infrastructure. Biological soil strengthening aims to maximize resource utilization, limit waste production, and improve overall sustainability by collaborating with natural systems and ecological processes. In the current study, from a series of UCS and CBR tests under varying curing methods and curing duration, it was found that freeze-drying GPA-BG biopolymer improves the engineering performance of unsuitable subgrade soil to a stabilized subgrade foundation for pavements, without the need for expensive foundations or chemical treatment, thus saving resources, with a cost reduction of 12.5%. Although thermal treatment in the field is a challenge over large areas, adequate initial air-curing is instrumental in the preservation of the strength of the stabilized soils.

## Figures and Tables

**Table 1:** Soil properties.

Properties	Low Compressible Clay	Low Compressible Silt
Specific gravity	2.74	2.71
Sand (%)	13	17
Clay (%)	54	36
Silt (%)	33	47
Liquid limit (%)	34	29
Plasticity index (%)	14.8	7.7
USCS	CL	ML
Coefficient of permeability (cm/s)	7.31 × 10^−5^	2.6 × 10^−4^
Optimum moisture content (OMC) (%)	8	10
Maximum dry unit (MDU) weight (kN/m^3^)	21.8	19.7
UCS (kPa)	196	217
Soaked CBR (%)	3.4	3.8
Unsoaked CBR (%)	7.3	8.1
pH	7.27	7.46

**Table 2:** Design thickness catalog for CBR = 9%; ML + 1% GPA-BG.

MSA	Modulus of Granular Base + Sub-Base (MPa)	Modulus of Subgrade (MPa)	Thickness of Dense Bituminous Macadam (mm)	Thickness of Granular Layer (mm)
5	135.44	62.41	140	200
10	138.45	62.41	160	210
15	135.44	62.41	180	200
20	141.38	62.41	190	220
25	141.38	62.41	195	220
30	142.81	62.41	205	225
35	139.92	62.41	210	215
40	135.44	62.41	220	200
45	127.54	62.41	225	175
50	125.89	62.41	230	170

**Table 3:** Cost comparison.

S. No.	CBR of Soil	Cost of Bituminous Surfacing (INR)	Cost of the Granular Layer (INR)	Transportation Cost of Granular Layer (INR 50/m^3^)	Cost of GPA-BG in the Subgrade (INR)	Total Cost (INR)
1	3.77%	2,353,754	969,373	45,938	NA	3,369,065
2	9.38%	2,032,788	870,458	42,281	688	2,945,870

## Data Availability

Data are contained within the article.
